# Supplementary material for: The within-subject application of diffusion tensor MRI and CLARITY reveals brain structural changes in Nrxn2 deletion mice
Source: Mol Autism. 2019 Feb 28;10:8. doi: 10.1186/s13229-019-0261-9 (PMC6394023; doi:10.1186/s13229-019-0261-9)
Supplement: Supplementary file 1 — Supplemental materials and methods (DOCX 6377 kb) [file 13229_2019_261_MOESM1_ESM.docx]

**Additional file 1**

**The within-subject application of diffusion tensor MRI and CLARITY reveals brain structural changes in *Nrxn2* deletion mice**

**Supplemental Material**

Eleftheria Pervolaraki Ph.D, Adam L. Tyson MRes, Francesca Pibiri Ph.D, Steven L. Poulter Ph.D, Amy C. Reichelt Ph.D, R. John Rodgers Ph.D, Steven J. Clapcote Ph.D, Colin Lever Ph.D, Laura C. Andreae M.D. Ph.D, James Dachtler Ph.D

**Supplemental Materials and Methods**

**Diffusion Tensor MRI**

**Data Acquisition**

Brain MR imaging was performed on a vertical 9.4 Tesla spectrometer (Bruker AVANCE II NMR, Ettlingen, Germany) with an 89 mm wide bore, 3 radio frequency channels with digital broadband frequency synthesis (6-620 MHz) and an imaging coil with diameter of 25 mm for hydrogen (1H). 3D images for each brain were obtained using a DT-MRI protocol (TE: 35 ms, TR: 700 ms, 10 signal averages). The field of view was set at 128 x 128 x 128, with a cubic resolution of 100 μm/pixel and a b value of 1200 s/mm^2^. For each brain, diffusion weighted images were obtained in 6 directions, based upon recent published protocols ([1-5](#_ENREF_1)). The subject of the number of diffusions gradients has been debated ([6](#_ENREF_6)), with studies suggesting limited benefits of using more than 6 directions in biological tissue ([7-9](#_ENREF_7)). The imaging time for each brain was 60 hours.

**CLARITY**

**Solutions:**

**Hydrogel solution:** 2% PFA 2% acrylamide 0.05% bis-acrylamide and 0.25% VA-044 thermal initiator (2,2’-Azobis[2-(2-imidazolin-2-yl) propane] dihydrochloride) in PBS, pH 7.4.

**Clearing buffer:** 8% Sodium dodecyl sulfate in 200mM boric acid, pH 8.5.

**Multiphoton imaging – methodological outline**

Cleared samples were mounted in custom 3D printed chambers for two-photon imaging. Images were acquired using ZEN Black (Zeiss, Germany). DAPI signal was detected using a 485 nm short pass filter, and neurofilament using a 500-550 nm band pass filter. The power of the excitation laser was varied to maximise the dynamic range for each image, but all other parameters were kept constant. The images were analysed using custom MATLAB (version 9.1, The Mathworks Inc.) scripts. Two-dimensional images were visualised using ImageJ ([10](#_ENREF_10)) and three-dimensional images using Vaa3D ([11](#_ENREF_11)).

**Multiphoton imaging and analysis – image analysis method**

**Pre-processing**

Image files were loaded into MATLAB (The Mathworks Ltd.) using the BioFormats toolbox ([12](#_ENREF_12)), and the raw image data were obtained along with the precise voxel dimensions from the metadata. Each two-dimensional (2D) image from the three-dimensional (3D) stack was initially corrected for uneven background illumination by element-wise division by a 2D reference image. This reference image was calculated as the mean 2D image through the 3D stack, which was smoothed using a 2D Gaussian kernel with a full-width at half maximum (FWHM) of 20 % of the geometric mean of the dimensions of the 2D image (mean dimension). The image was denoised by filtering the image with a Gaussian kernel with a standard deviation of one pixel. Background subtraction was carried out by subtracting a smoothed, filtered image (FWHM 10 % of the mean dimension). Each pixel was then smoothed using a 3D Gaussian kernel with FWHM of 1.5 μm (the largest axonal diameter expected according to Perge et al. ([13](#_ENREF_13)).

**Segmentation**

The numerical gradient of the image in each dimension $\left( \Delta X, \Delta Y, \Delta Z \right)$was calculated, and these were combined to calculate the magnitude of the gradient $\left( \sqrt{{\Delta X}^{2}+ {\Delta Y}^{2}+ {\Delta Z}^{2}} \right).$The resulting image was thresholded, using a combination of the Otsu (1979) and Rosin (2001) methods (Rosin threshold + 2/5 Otsu threshold) ([14](#_ENREF_14), [15](#_ENREF_15)). The gradient image highlights the edge of each axon; to combine these into a single object, the image was dilated and then eroded with a cubic structuring element (each side being 1.5 μm, to ‘close’ the largest axons as per Perge et al. ([13](#_ENREF_13))). Very small objects (less than 50 μm^3^) were removed from the image as they reflected noise, very small neuronal processes and gradients around cells.

Owing to variations in staining intensity of different axons, the thresholding produced segmented axons of various thicknesses that did not necessarily reflect the true structure. To remove this bias, the thresholded image was skeletonised using a homotopic thinning algorithm ([16](#_ENREF_16)) implemented in MATLAB([17](#_ENREF_17)). The resulting image was dilated and then eroded using a cubic structuring element (10 pixels on each side for dilation, 9 for erosion) to produce connected processes with a uniform two-pixel diameter. This dilation ensured that the voxels in the binary image were connected via their faces (6-connected) rather than just their corners (26-connected), which better reflects the true structure of biological processes. This method detects most large axons at the expense of smaller processes, and the loss of any information about axon diameter. These steps are outlined in Supp. Figure 2.

**Analysis**

The density of axons was calculated as the fraction of the image volume taken up by the segmented axons. A measure of axonal alignment was calculated by determining the mean axonal alignment along each dimension. This alignment was calculated by moving along the 3D image in a single dimension, keeping the coordinates in the other dimensions constant, and counting the number of times the pixel intensity did not change (i.e. how many times an axon was not entered or left). This number was averaged across each face of the image volume and scaled to the length of each dimension to produce a metric of how constant the image intensity is in that dimension. The perfect case of no intensity change (i.e. all axons are aligned perfectly with a particular dimension) gives a value of 1. The greater the difference between this measure in each three dimensions, the more aligned the axons must be (i.e. their directions are anisotropic). The standard deviation of this measure across the three dimensions was calculated as the axonal alignment.

The alignment calculation is illustrated in Supp. Figure 3 for a simple, two-dimensional case. Supp. Figure 3a shows the case of low axonal alignment, and Supp. Figure 3b shows the case of high axonal alignment. In each case, for illustration, each pixel represented by a small square on the grid is classed as either containing an axon or not. In the real images, the pixels are smaller, and are actually binary. In each axis, the number of pixel transitions in which the presence of an axon does not change is divided by the number of transitions, and the average is calculated. The standard deviation of this average for all axes is the measure of axonal alignment. When the alignment is low, the two averages are similar, and the standard deviation is low. When the alignment is high the two averages are very different, and the standard deviation is high. To analyse the real data, this same calculation is carried out in 3D, but in a much larger grid of voxels. Cell density was calculated as the number of cells per mm^3^.

**Table S1**

| **Brain Region** | **DTI Measure** | **ANOVA Comparison** | **F Value** | **P Value** |
| --- | --- | --- | --- | --- |
| Amygdala-Anterior Hippocampus | AD | Genotype | F_(1,10)_ <1 | P = 0.164 |
|  |  | Hemisphere | F_(1,10)_ = 2.10 | P = 0.097 |
|  |  | **Genotype x Hemisphere** | **F_(1,10)_ = 12.12** | **P = 0.023** |
| Amygdala-Anterior Hippocampus | RD | Genotype | F_(1,10)_ <1 | P = 0.149 |
|  |  | Hemisphere | F_(1,10)_ = 1.32 | P = 0.106 |
|  |  | Genotype x Hemisphere | F_(1,10)_ <1 | P = 0.155 |
| Amygdala-Posterior Hippocampus | AD | Genotype | F_(1,10)_ <1 | P = 0.142 |
|  |  | Hemisphere | F_(1,10)_ <1 | P = 0.189 |
|  |  | Genotype x Hemisphere | F_(1,10)_ = 4.54 | P = 0.061 |
| Amygdala-Posterior Hippocampus | RD | Genotype | F_(1,10)_ <1 | P = 0.151 |
|  |  | Hemisphere | F_(1,10)_ <1 | P = 0.135 |
|  |  | Genotype x Hemisphere | F_(1,10)_ <1 | P = 0.128 |
| BLA-Anterior Hippocampus | AD | Genotype | F_(1,10)_ <1 | P = 0.167 |
|  |  | **Hemisphere** | **F_(1,10)_ = 6.59** | **P = 0.047** |
|  |  | **Genotype x Hemisphere** | **F_(1,10)_ = 10.53** | **P = 0.032** |
| BLA-Anterior Hippocampus | RD | Genotype | F_(1,10)_ <1 | P = 0.158 |
|  |  | Hemisphere | F_(1,10)_ = 2.59 | P = 0.092 |
|  |  | Genotype x Hemisphere | F_(1,10)_ <1 | P = 0.173 |
| BLA-Posterior Hippocampus | AD | Genotype | F_(1,10)_ <1 | P = 0.169 |
|  |  | **Hemisphere** | **F_(1,10)_ = 12.79** | **P = 0.018** |
|  |  | **Genotype x Hemisphere** | **F_(1,10)_ = 12.97** | **P = 0.02** |
| BLA-Posterior Hippocampus | RD | Genotype | F_(1,10)_ <1 | P = 0.162 |
|  |  | Hemisphere | F_(1,10)_ = 3.11 | P = 0.077 |
|  |  | Genotype x Hemisphere | F_(1,10)_ <1 | P = 0.178 |

Statistical analysis of the anterior (Bregma -1.94 mm), and posterior (Bregma -3.28 mm) amygdala-hippocampal connections, analysed for axial diffusion (AD) and radial diffusion (RD). Analysis was performed using repeated measure two-way ANOVAs for genotype and hemisphere (Benjamini-Hochberg corrected (corrected P values stated)).

**Table S2**

| **Brain Region** | **CLARITY Measure** | **ANOVA Comparison** | **F Value** | **P Value** |
| --- | --- | --- | --- | --- |
| M1 | OI | Genotype | F_(1,10)_ <1 | P = 0.182 |
|  |  | Hemisphere | F_(1,10)_ = 1.74 | P = 0.108 |
| M1 | Cell Density | Genotype | F_(1,10)_ = 2.04 | P = 0.099 |
|  |  | Hemisphere | F_(1,10)_ = 1.41 | P = 0.117 |
| M1 | Fibre Density | Genotype | F_(1,10)_ <1 | P = 0.171 |
|  |  | Hemisphere | F_(1,10)_ <1 | P = 0.176 |
| S1 | OI | Genotype | F_(1,10)_ <1 | P = 0.185 |
|  |  | **Hemisphere** | **F_(1,10)_ = 36.86** | **P = 0.005** |
| S1 | Cell Density | Genotype | F_(1,10)_ <1 | P = 0.131 |
|  |  | **Hemisphere** | **F_(1,10)_ = 13.73** | **P = 0.016** |
| S1 | Fibre Density | Genotype | F_(1,10)_ = 1.73 | P = 0.110 |
|  |  | **Hemisphere** | **F_(1,10)_ = 8.51** | **P = 0.038** |
| BF | OI | Genotype | F_(1,10)_ <1 | P = 0.191 |
|  |  | **Hemisphere** | **F_(1,10)_ = 10.59** | **P = 0.034** |
| BF | Cell Density | Genotype | F_(1,10)_ <1 | P = 0.133 |
|  |  | **Hemisphere** | **F_(1,10)_ = 8.70** | **P = 0.041** |
| BF | Fibre Density | Genotype | F_(1,10)_ <1 | P = 0.144 |
|  |  | Hemisphere | F_(1,10)_ <1 | P = 0.126 |

Statistical analysis of the primary motor cortex (M1), primary somatosensory cortex (S1) and the barrel field (BF). CLARITY imaged regions were then analysed for orientation index (OI), cell density and fibre density. Analysis was performed using repeated measure two-way ANOVAs for genotype and hemisphere (Benjamini-Hochberg corrected (corrected P values stated)).

**Figure S1**

**
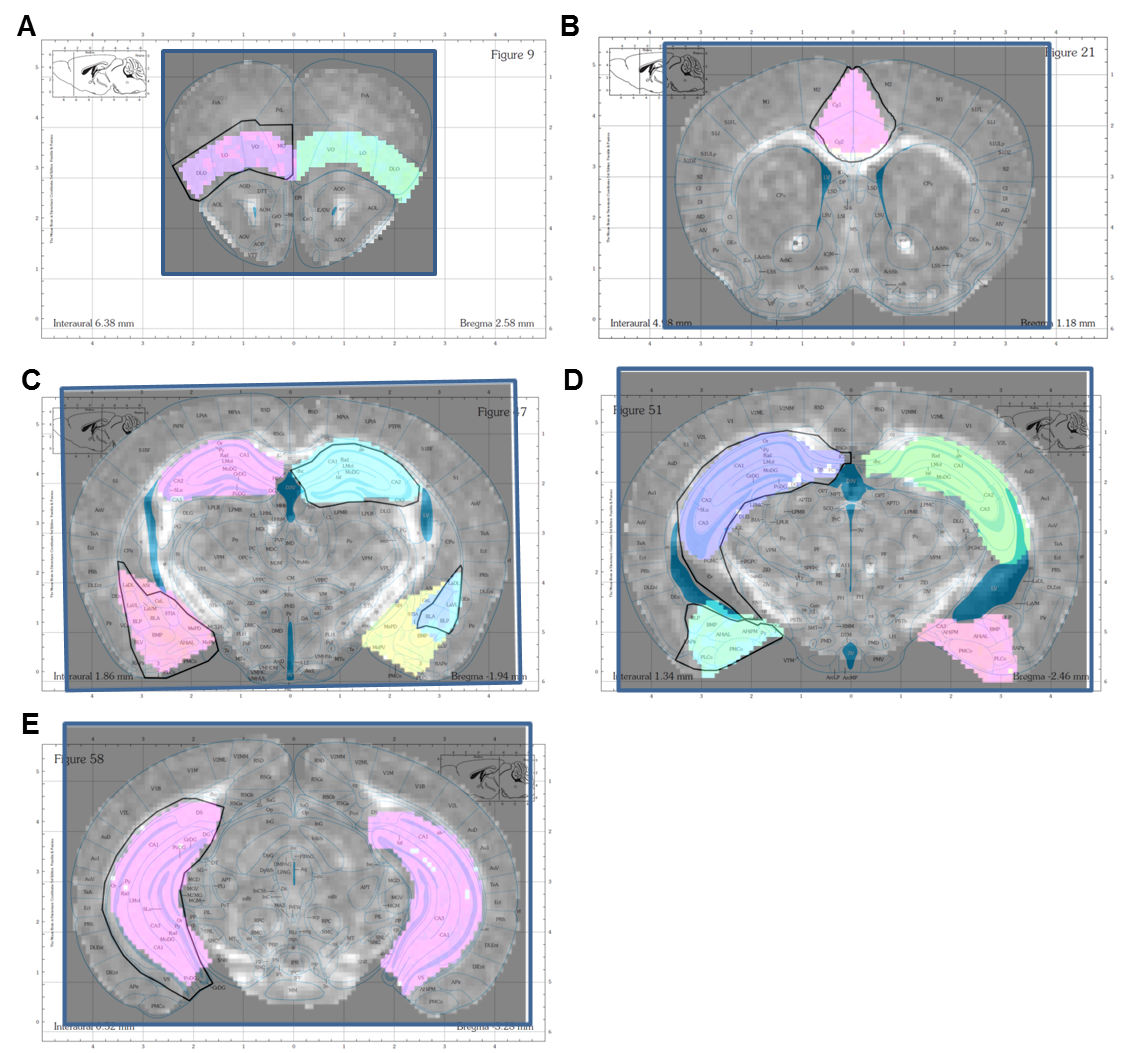
**

Atlas maps representing manual segmentation of regions of interest (ROI), overlaid with segmented brain regions from a fractional anisotropy-coloured brain slice. (**A**) The orbitofrontal cortex ROI. (**B**) The ACC ROI. (**C**) The anterior hippocampus, anterior amygdala and basolateral amygdala ROI. (**D**) The mid hippocampus and posterior amygdala ROI. (**E**) The posterior hippocampus ROI. The atlas maps were used with the permission of the Authors ([18](#_ENREF_18)).

**Figure S2**

**
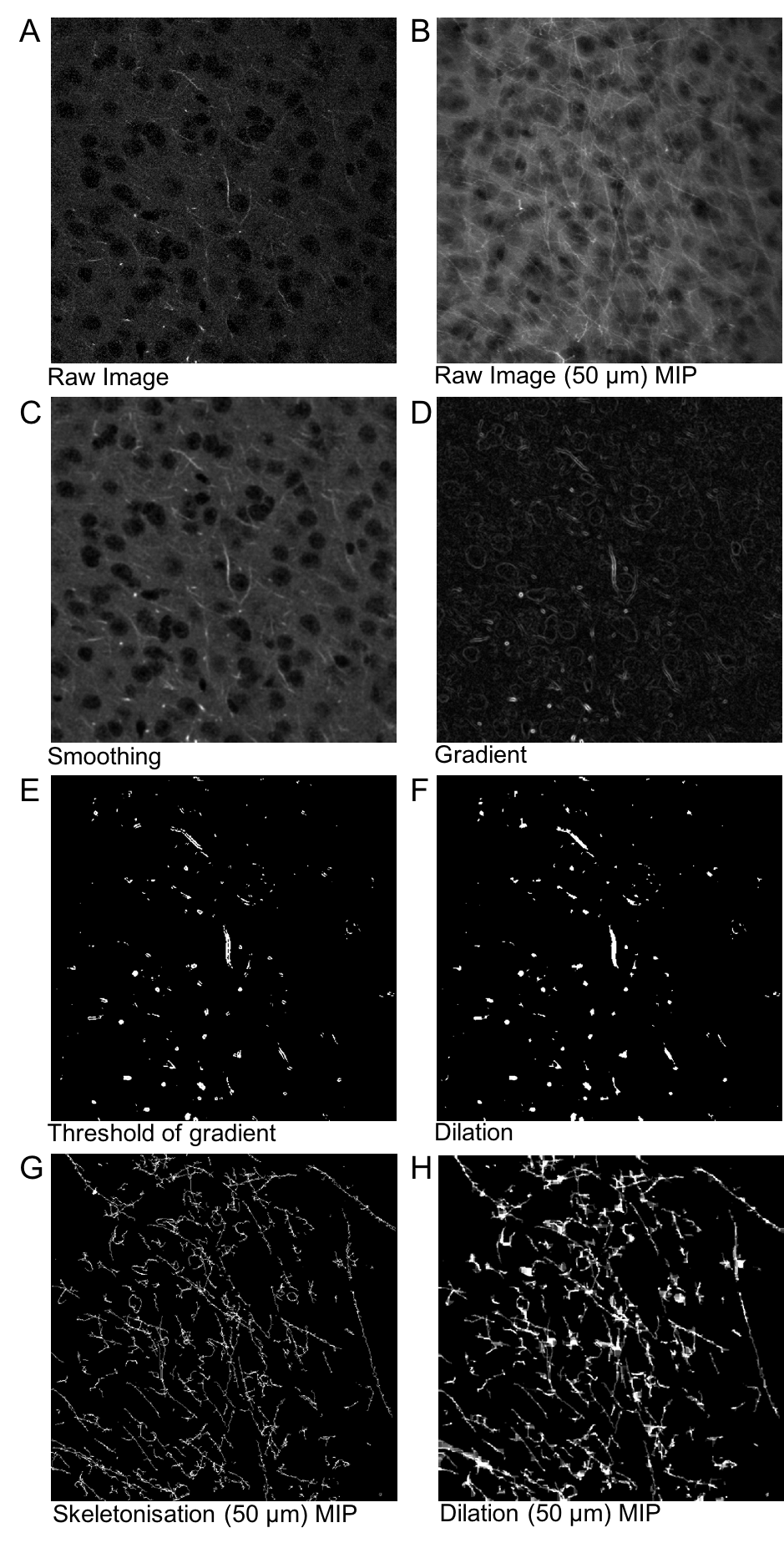
**

Analysis methodology of axonal segmentation from multiphoton images.

**Figure S3**


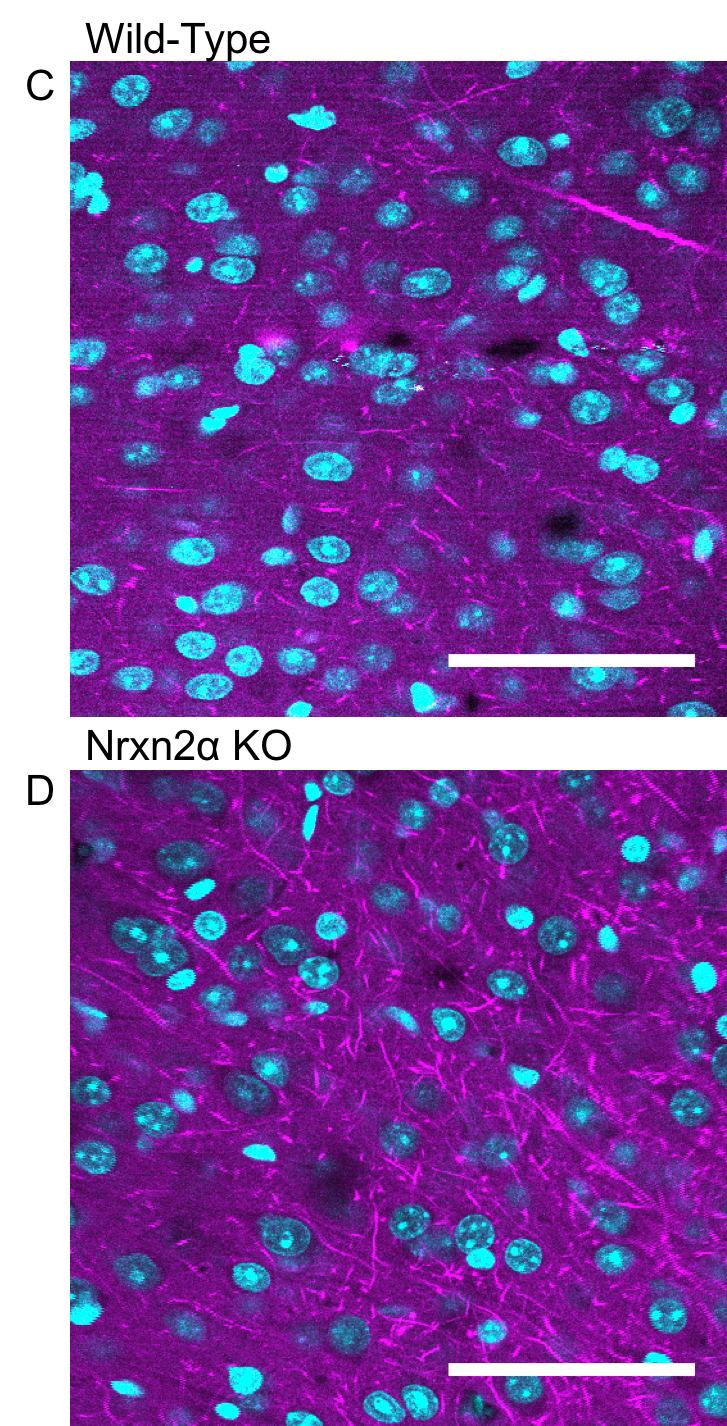
**
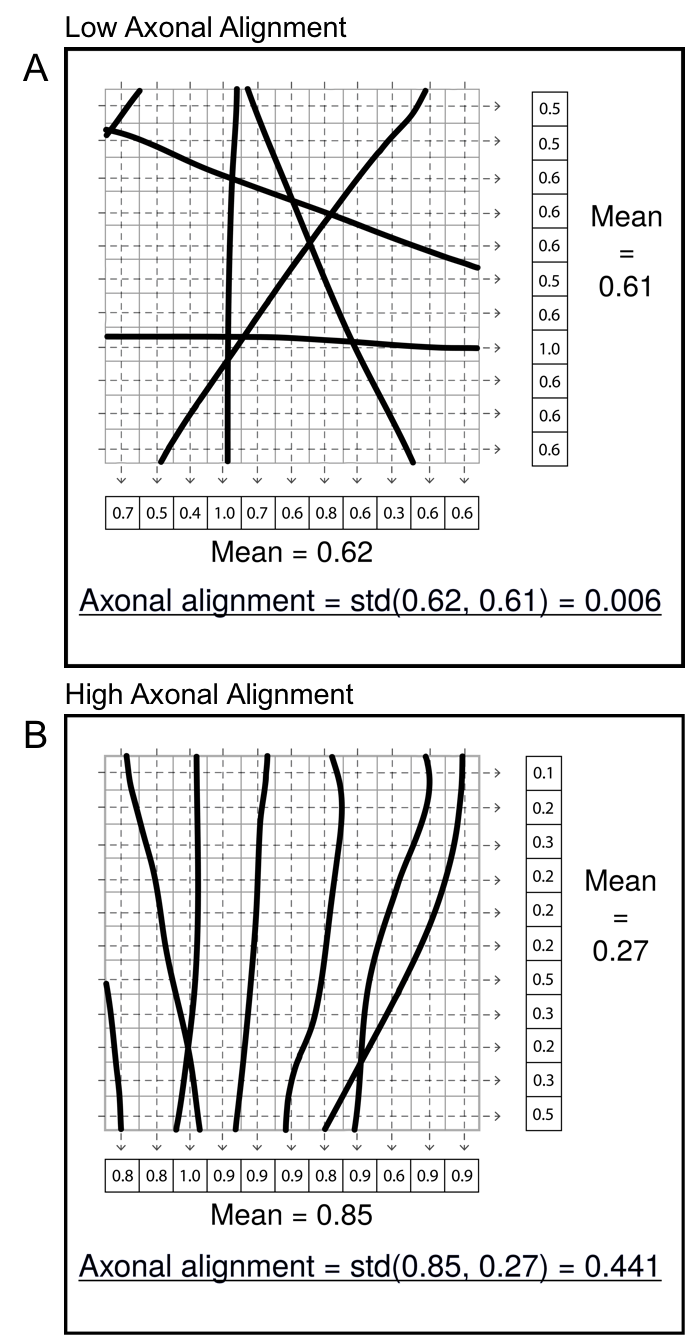
**

**A and B.** Illustration of the axonal alignment calculation in a simple two-dimensional case. The grey grid represents the image pixels, the black lines axons and the dashed lines represent the calculation process. Standard deviation denoted as std. **C and D.** Cingulate cortex images taken from wild-type and Nrxn2α KO mice, visually representing the greater axonal alignment and density in KO mice.

**Figure S4**


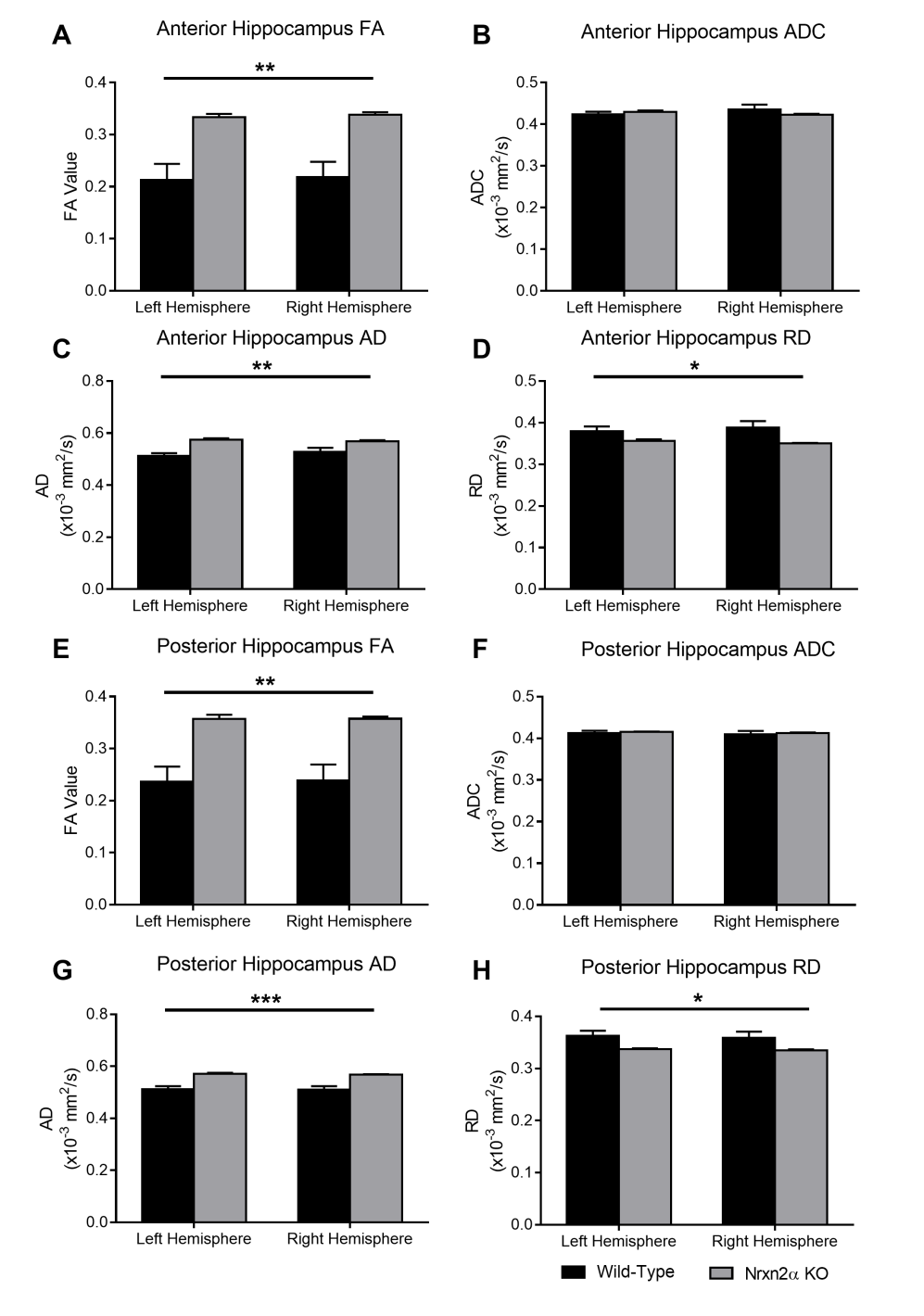


DTI quantified for the whole anterior hippocampus (Bregma -1.06 mm – -2.46 mm) and posterior hippocampus (Bregma -2.54 mm – -3.16 mm). (**A**) Fractional anisotropy (FA) in the anterior hippocampus was significantly increased in Nrxn2α KO mice (genotype: F_(1,10)_ = 15.63, p = 0.0027) but (**B**) apparent diffusion coefficient (ADC) was not altered (genotype: F_(1,10)_ <1, p = 0.738). (**C**) Axial diffusivity (AD) (genotype: F_(1,10)_ = 16.17, p = 0.0024) and (**D**) radial diffusivity (RD) (genotype: F_(1,10)_ = 5.05, p = 0.048) were both significantly altered in Nrxn2α KO mice. In the posterior hippocampus, in Nrxn2α KO mice, (**E**) FA was significant increased (genotype: F_(1,10)_ = 15.62, p = 0.0027), (**F**) ADC was similar to wild-types (genotype: F_(1,10)_ <1, p = 0.679), (**G**) AD was increased (genotype: F_(1,10)_ = 22.31, p = 0.0008) and (**H**) RD was significantly reduced (genotype: F_(1,10)_ = 5.34, p = 0.043). Error bars represent s.e.m. * = P<0.05, ** = P<0.01, *** = P<0.001. Wild-type n=6, Nrxn2α KO n=6.

**Figure S5**


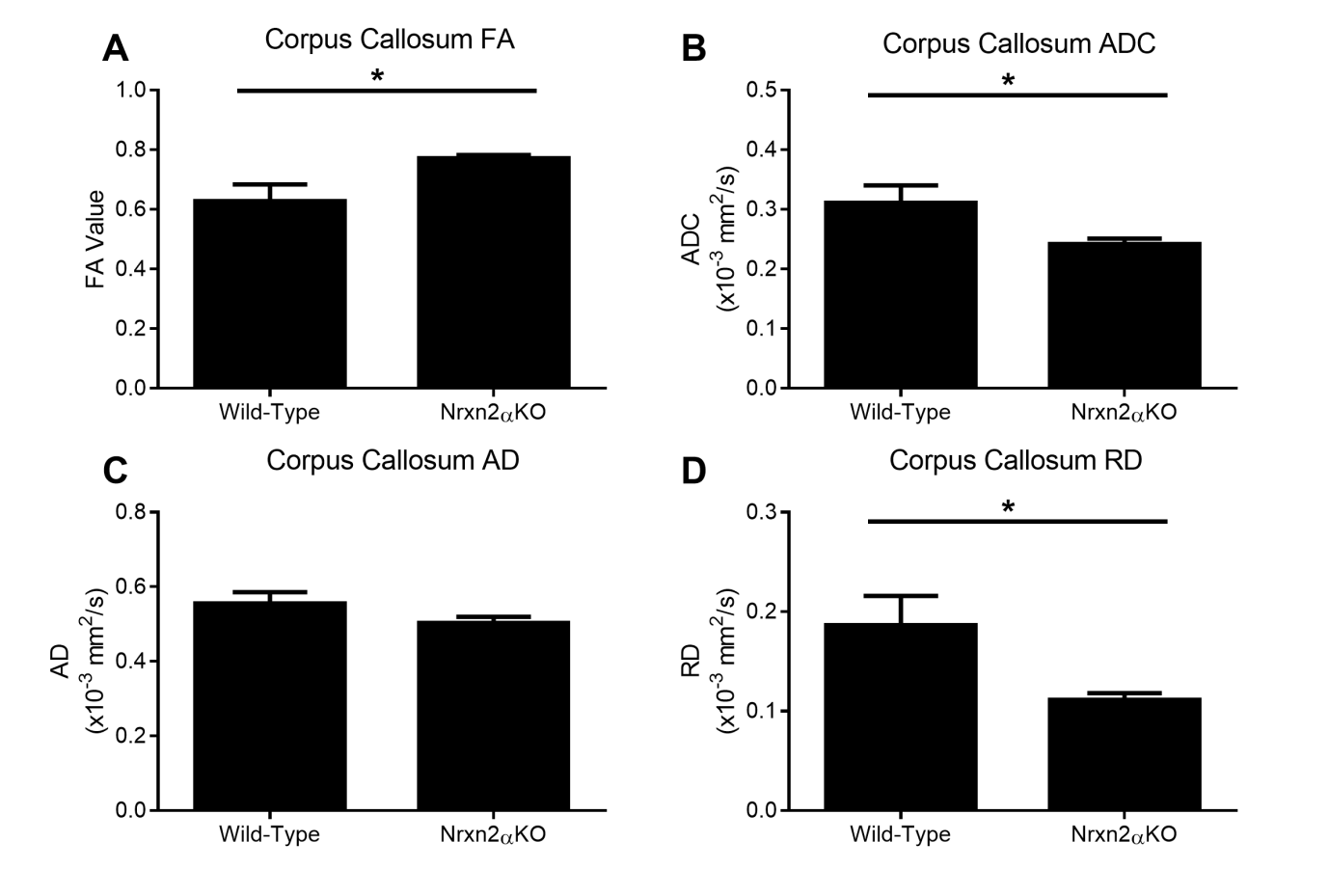


DTI quantification of the corpus callosum (Bregma 0.98 mm). To examine the integrity of white matter tracts within Nrxn2α KO mice, we examined diffusivity in the corpus callosum. (**A**) Fractional anisotropy (FA) was significantly increased in Nrxn2α KO mice (genotype: t_(10)_ = 2.50, p = 0.032) and apparent diffusion coefficient (ADC) (**B**) significantly decreased (genotype: t_(10)_ = 2.28, p = 0.046). This difference appeared to be driven predominantly by radial diffusivity (RD), as axial diffusion (AD) (**C**) was not significantly different (genotype: t_(10)_ = 1.49, p = 0.168) whilst RD (**D**) was significantly reduced in Nrxn2α KO mice (genotype: t_(10)_ = 2.45, p = 0.034). Error bars represent s.e.m. * = P<0.05. Wild-type n=6, Nrxn2α KO n=6.

**Figure S6**

**
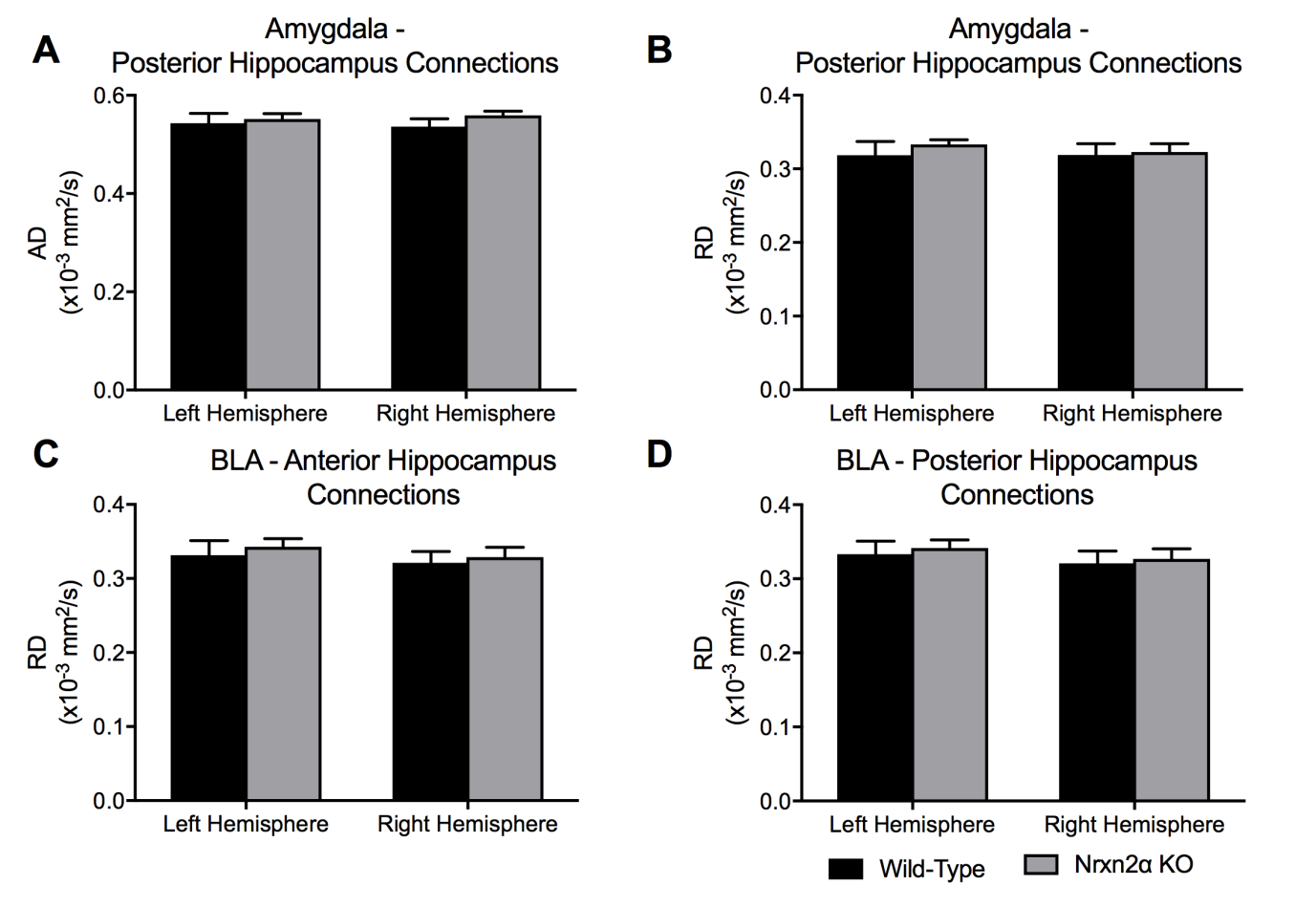
**

Axial diffusivity (AD) and radial diffusivity (RD) of computed tracts of connections from the amygdala to hippocampus. Tracts from the anterior amygdala to the posterior hippocampus (Bregma -2.46 mm) were analysed for AD (**A**) and RD (**B**). No significant differences between the tracts of Nrxn2α KO mice were observed. No significant differences were found for RD of tracts specifically from the basolateral nuclei of the amygdala (BLA) to the anterior (**C**) or posterior (**D**) hippocampus. Error bars represent s.e.m. Wild-type n=6, Nrxn2α KO n=6.

**Figure S7**


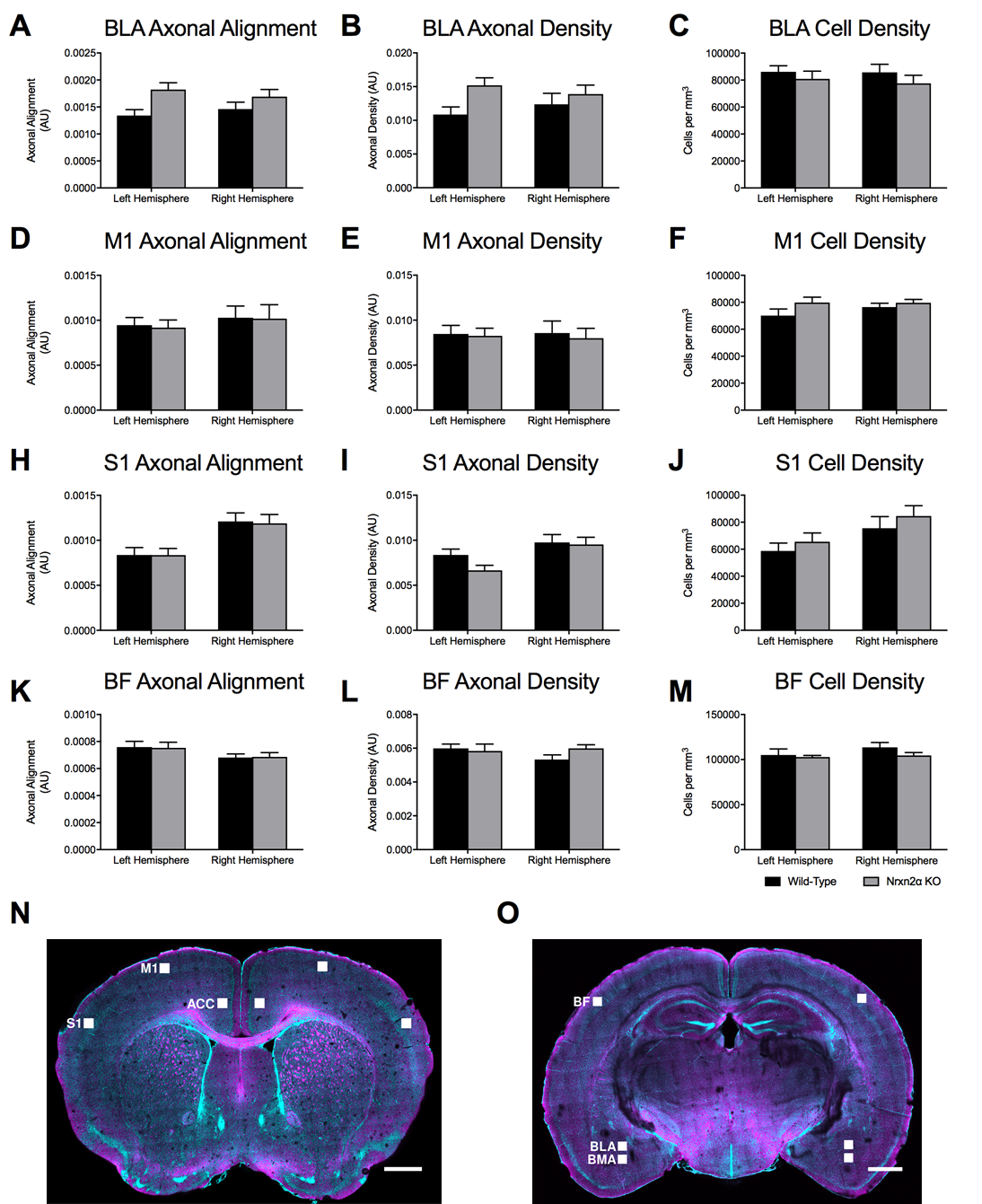


CLARITY-derived quantification of fibres and cell density within the basolateral amygdala (BLA) and control regions (**A**) Although there were trends towards increased axonal alignment and fibre density (**B**) in Nrxn2α KO mice, no significant differences were found. (**C**) Cell density in the BLA was similar between the genotypes. Statistical analysis (Supp. Table 4) was performed for the primary motor cortex (M1; **D-F**), primary somatosensory cortex (S1; **H-J**) and the barrel field (BF; **K-M**). No genotypic differences were found for any measure within these cortical regions. (N-O) CLARITY images of the scanned regions of interest. Error bars represent s.e.m. Wild-type n=6, Nrxn2α KO n=6.

**References**

1. Wu D, Xu J, McMahon MT, van Zijl PC, Mori S, Northington FJ, et al. In vivo high-resolution diffusion tensor imaging of the mouse brain. NeuroImage. 2013;83:18-26.

2. Vo A, Sako W, Dewey SL, Eidelberg D, Ulug AM. 18FDG-microPET and MR DTI findings in Tor1a+/- heterozygous knock-out mice. Neurobiology of disease. 2015;73:399-406.

3. Kumar M, Duda JT, Hwang WT, Kenworthy C, Ittyerah R, Pickup S, et al. High resolution magnetic resonance imaging for characterization of the neuroligin-3 knock-in mouse model associated with autism spectrum disorder. PloS one. 2014;9(10):e109872.

4. Ruest T, Holmes WM, Barrie JA, Griffiths IR, Anderson TJ, Dewar D, et al. High-resolution diffusion tensor imaging of fixed brain in a mouse model of Pelizaeus-Merzbacher disease: comparison with quantitative measures of white matter pathology. NMR in biomedicine. 2011;24(10):1369-79.

5. Kim S, Pickup S, Fairless AH, Ittyerah R, Dow HC, Abel T, et al. Association between sociability and diffusion tensor imaging in BALB/cJ mice. NMR in biomedicine. 2012;25(1):104-12.

6. Jones DK. The effect of gradient sampling schemes on measures derived from diffusion tensor MRI: a Monte Carlo study. Magnetic resonance in medicine. 2004;51(4):807-15.

7. Lebel C, Benner T, Beaulieu C. Six is enough? Comparison of diffusion parameters measured using six or more diffusion-encoding gradient directions with deterministic tractography. Magnetic resonance in medicine. 2012;68(2):474-83.

8. Ni H, Kavcic V, Zhu T, Ekholm S, Zhong J. Effects of number of diffusion gradient directions on derived diffusion tensor imaging indices in human brain. AJNR American journal of neuroradiology. 2006;27(8):1776-81.

9. Hasan KM, Parker DL, Alexander AL. Comparison of gradient encoding schemes for diffusion-tensor MRI. Journal of magnetic resonance imaging : JMRI. 2001;13(5):769-80.

10. Schindelin J, Arganda-Carreras I, Frise E, Kaynig V, Longair M, Pietzsch T, et al. Fiji: an open-source platform for biological-image analysis. Nature methods. 2012;9(7):676-82.

11. Peng H, Ruan Z, Long F, Simpson JH, Myers EW. V3D enables real-time 3D visualization and quantitative analysis of large-scale biological image data sets. Nature biotechnology. 2010;28(4):348-53.

12. Linkert M, Rueden CT, Allan C, Burel JM, Moore W, Patterson A, et al. Metadata matters: access to image data in the real world. The Journal of cell biology. 2010;189(5):777-82.

13. Perge JA, Niven JE, Mugnaini E, Balasubramanian V, Sterling P. Why do axons differ in caliber? The Journal of neuroscience : the official journal of the Society for Neuroscience. 2012;32(2):626-38.

14. Otsu N. A Threshold Selection Method from Gray-Level Histograms. IEEE Transactions on Systems, Man, and Cybernetics. 1979;9(1):62-6.

15. Rosin P. Unimodal thresholding. Pattern Recognition. 2001;34(11):2083-96.

16. Lee T, Kashyap R, Chu C. Building Skeleton Models via 3-D Medial Surface Axis Thinning Algorithms. CVGIP: Graphical Models and Image Processing. 1994;56(6):462-78.

17. Kerschnitzki M, Kollmannsberger P, Burghammer M, Duda GN, Weinkamer R, Wagermaier W, et al. Architecture of the osteocyte network correlates with bone material quality. Journal of bone and mineral research : the official journal of the American Society for Bone and Mineral Research. 2013;28(8):1837-45.

18. Franklin K, Paxinos G. The Mouse Brain in Stereotaxic Coordinates: Academic Press; 2008.
